# Examining time–frequency mechanisms of full-fledged deep sleep development in newborns of different gestational age in the first days of their postnatal development

**DOI:** 10.1038/s41598-022-26111-3

**Published:** 2022-12-14

**Authors:** Anton R. Kiselev, Oxana M. Drapkina, Mikhail Yu. Novikov, Olga S. Panina, Yuri V. Chernenkov, Maksim O. Zhuravlev, Anastasiya E. Runnova

**Affiliations:** 1 National Medical Research Center for Therapy and Preventive Medicine, 10(3) Petroverigsky Pereulok, Moscow, Russia 101990; 2 Saratov State Medical University, Saratov, Russia; 3 Saratov State University, Saratov, Russia

**Keywords:** Paediatric research, Sleep

## Abstract

Early age-related changes in EEG time–frequency characteristics during the restful sleep of newborns of different gestational ages result in the development of conventional EEG signs of deep sleep already during the first postnatal week of their life. Allocating newborns to different groups based on their gestational age and duration of postnatal period allowed demonstrating substantial intergroup differences in brain activity during sleep and wakefulness, along with significant variability in the time–frequency characteristics of brain activity. The process of conventional deep sleep development in infants born prior to the week 35 of gestation is associated with an increase in the power of alpha activity in the sensorimotor cortex of the brain.

## Introduction

Currently, studying human sleep is among the most important topics that unite neuroscientists, physiologists, physicists, and information technology specialists^1–5^. Strong interest in this scientific field is due to the fact that early diagnosis and treatment of sleep disorders in patients could overcome diseases leading to neurological disorders^3,4^ and improve the quality of life in patients. One of the main tools for diagnosing sleep disorders is polysomnography^5^. Analysis of polysomnography results allows identifying stages of sleep and their durations, along with examining the correlation between the dynamics of various physiological parameters and sleep stages. Accurate determination of the sleep disorder mechanism contributes to choosing the most successful treatment method, thereby preventing further unfavorable course of the disease^4^. At the same time, there is no agreement among scientists in understanding the fundamental issue of the vital role of full-fledged sleep in providing normal body functioning. Same is true about objective characteristics of the functional activity of both brain and cardiovascular system, making it possible to automatically predict the imminent onset of sleep and stages of nocturnal and diurnal sleep with a high accuracy.

Even though the mysteries of sleep in adults are not solved yet, special attention of scientists is attracted by examining brain functioning at the very beginning of ontogenesis, i.e., in the first days of life in newborns. Given poorly developed response of sensory systems during this period, it becomes especially important to analyze the structure of brain activity during the transition from sleep to wakefulness and accompanying changes in the objective characteristics of the brain. At present, in order to analyze the change in brain activity of newborns, scientists use various imaging techniques to record brain activity and examine the recorded data by means of various analytical procedures. Published studies, analyzing magnetic resonance imaging (MRI) of developing neonatal brain via automatic segmentation^6^, evaluated diffusion tensor imaging (DTI) of white and gray matter in the fetal, neonatal, and infant brains by using structure determination and reconstruction^6,7^. Other published studies examined the development of the brain during the first year of life via analyzing MRI images using the theory of finite deformations^8^, and analyzed anomalies in brain development of newborns with congenital heart defects using the statistical analysis of MRI and DTI^9^.

Electroencephalography (EEG) signal is most often used as a characteristic of brain activity, which is associated with a low cost and ease of implementation of such type of recording. Analysis of EEG signal variations also helps in deciphering varying brain development processes in newborns, including those born prematurely. Such interest is caused by fundamental awareness in the problem of human brain development, as well as by the necessity of early clinical detection of brain injury risk and early treatment aimed to reduce neurological dysfunctions. Quantifying brain health could maximize the utility of existing monitoring technologies in the neonatal intensive care unit (NICU). An immense interdisciplinary interest in this scientific field highlights the potential of a quantitative approach to improving clinical outcomes in children^10–15^. Newborns undergo multiple physiological changes during the transition from intrauterine to independent life, the pathological dynamics of which (e.g., hemodynamic instability) could lead to serious brain damage. The incidence of the latter reaches 1 in 3 in infants born prior to the week 32 of gestation^16^. Brain function monitoring can help clinicians identifying infants at risk of injury and, if necessary, refining treatment strategies.

In contemporary clinical practice, EEG analysis in NICU, as well as in intensive care units for adult patients, actively employs the method of amplitude-integrated EEG (aEEG), based on a compressed one-dimensional representation of a long-term recording of two or more EEG channels, which allows promptly and relatively objectively drawing a conclusion about the current state of cerebral function and its dynamics via detecting typical patterns on the aEEG trend. Besides, aEEG is used to develop fully automated methods for EEG analysis, for example, in assessing brain maturation^15,17^ and classifying sleep states^14,18^. Such systems can be integrated into an incubator/baby cot, thereby continuously providing a doctor without an expert knowledge of functional monitoring with dynamic information about the state of brain functions.

At the same time, the use of aEEG obviously represents a significant simplification of brain activity processes, reducing the complex multi-frequency distributed oscillatory dynamics of brain electrical potentials to the average characteristic obtained from spatially distributed electrode points. Thus, even though characteristic aEEG patterns have been quite successfully identified for critical and convulsive states of preterm infants, the markers of normal development processes of brain electrical activity in the immediate postnatal period in full-term and premature infants remain largely unknown. Many environmental factors, including the process of childbirth, may have a significant impact on early postnatal adaptation^19–21^. Existing EEG studies demonstrated that changes in some characteristics during this period are specific to different gestational ages^22–24^. However, a small number of nonspecific EEG features used in these studies (e.g., without characteristics of spatial and temporal organization) are not sufficient to capture the full complexity of the EEG in the first postnatal days. Besides, there is no quantitative assessment of changes in qualitative characteristics over time.

Currently, for newborns, in addition to the state of active wakefulness (AW), usually just two stages of sleep are distinguished: quiet sleep (QS) and active sleep (AS)^25–28^. The QS is characterized by substantial changes in the aEEG pattern, tonic EMG, regular respiration, and no body movements. The AS state is characterized by irregular respiratory pattern, rapid eye movement (REM), and repetitive aEEG patterns. However, QS and AS phases, in general, are not direct equivalents of NREM (deep sleep) and REM sleep stages in adults^29,30^. Besides, it is generally believed that full-fledged deep sleep develops in children by the third or fourth month of their postnatal life, which, to a certain extent, can be a marker of successful neurological development of a child^31,32^.

Our study includes examining an emergence and primary dynamics of deep sleep markers in newborns of different gestational ages in the first week of their life from the standpoint of traditional time–frequency analysis. In addition, we review the possibility of creating a system for a unified objective detection and assessment of the sleep quality in newborns based on automatic EEG analysis.

## Results

We present the results of a numerical analysis of various oscillatory activity detected via EEG monitoring recorded in full-term and late premature newborns, distributed among two groups based on their gestational age. Groups I and II included newborns with gestational age of 38–41 (N^I^ = 50) and 34–36 weeks (N^II^ = 48), respectively. Detailed information about patient groups is provided in the *Patients* section and Table 1. All newborns underwent the complex noninvasive procedures of recording various biomedical signals, specifically EEG, EOG, ECG, EMG Left Arm, and EMG Right Leg. In group I, neurophysiological monitoring was performed twice, 8 h and 10 days after birth. Newborns from group II underwent similar monitoring solely on day 10 after their birth. For convenience of further presentation of the results, we use the following notation for the above-mentioned recordings: GA38–41^8h^ and GA38–41^10d^ in group I, and GA34–37^10d^ in group II.

**Table 1:** Characteristics of newborns in groups I and II.

Parameters	Group	Mean	Standard deviation	Min.	Median	Max.	Q3	Q1
Mean gestational age at birth, weeks	(Group I, N = 50)	39.00	1.06	37.96	38.37	41.22	39.72	38.16
	(Group II, N = 48)	35.23	0.77	33.76	35.22	36.91	35.84	34.59
Weight, g	(Group I)	3026.30	158.10	2750	3039.50	3350	3124	2906
	(Group II)	1786.77	183.71	1500	1808.50	2100	1938	1623
Mean age of mother, years	(Group I)	29.82	3.57	23.98	29.17	35.74	32.72	26.85
	(Group II)	32.09	3.38	26.42	31.73	37.47	35.01	29.35

Clinical neonatologists distinguish three main stages of sleep and wakefulness that are routinely used for the polysomnography analysis of newborns^28^, viz.: (1) quiet sleep (QS), (2) active sleep (AS), and (3) active wakefulness (AW).

**Figure 1:**
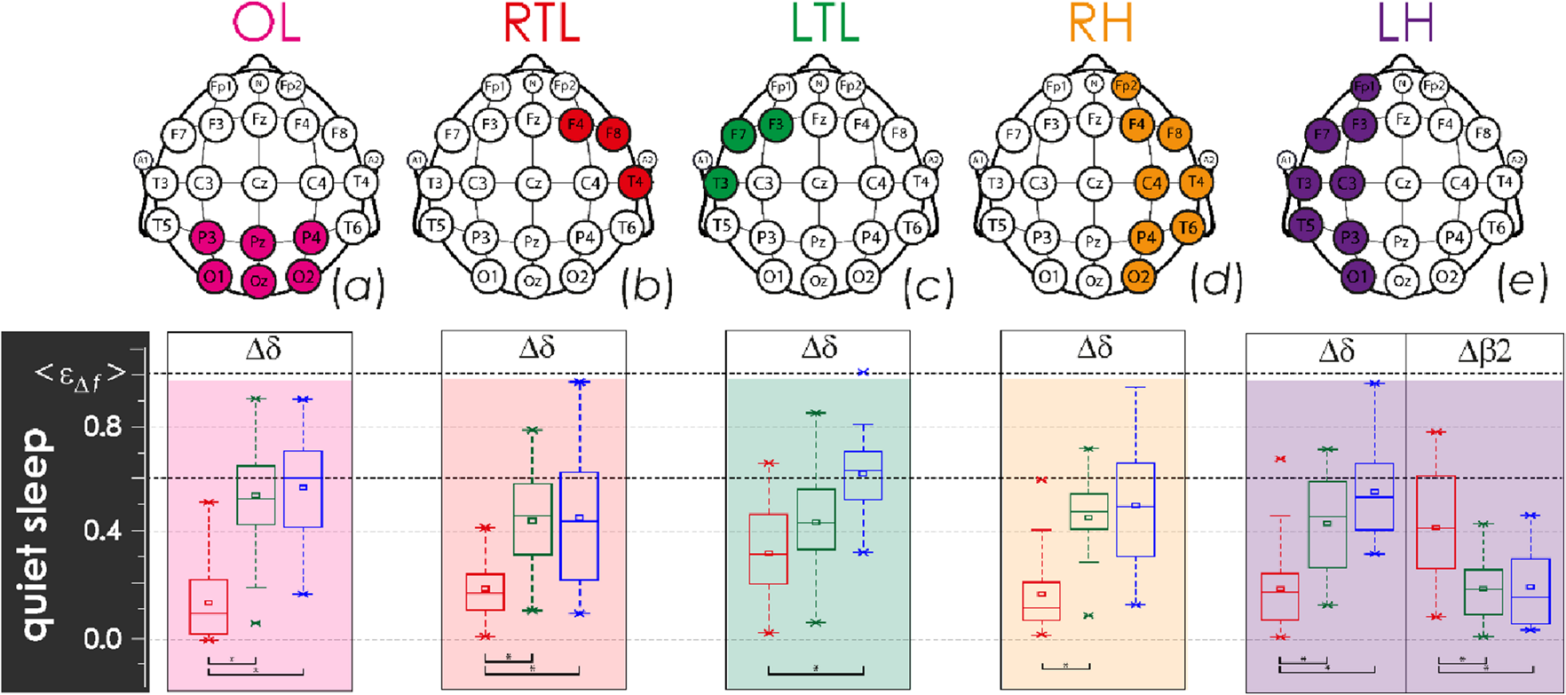
Distributions of the integral averages of relative CWT amplitudes, ${\langle {\upvarepsilon }_{\Delta \mathrm{f}}\rangle }_{\mathrm{QS}},$ based on EEG monitoring in newborn groups I and II for the various lobes of the brain, calculated under the physiological state of quiet sleep (QS): (**a**) diagrams for the occipital lobe (OL); (**b**) diagrams for the right temporal lobe (RTL); (**c**) diagrams for the left temporal lobe (LTL); (**d**) diagrams for the right hemisphere (RH); (**e**) diagrams for the left hemisphere (LH). The localization of EEG channels on the scalp for each brain lobe is shown above. Red color indicates estimates for group II (EEG monitoring event GA34–37^10d^), green and blue colors code the estimates of the first and second monitoring events in group I, GA38–41^8h^ and GA38–41^10d^, respectively. The diagrams depict the following statistical characteristics of numerical indicators: the first and the third quartiles (25–75%, inside the box), the median and the mean (transverse line and point inside the box, respectively), 1.5 interquartile ranges (shown by whiskers), and outliers represented by asterisks. At the bottom, underlining with asterisks highlights the pairs of values meeting the Mann–Whitney test with p < 0.001.

**Figure 2:**
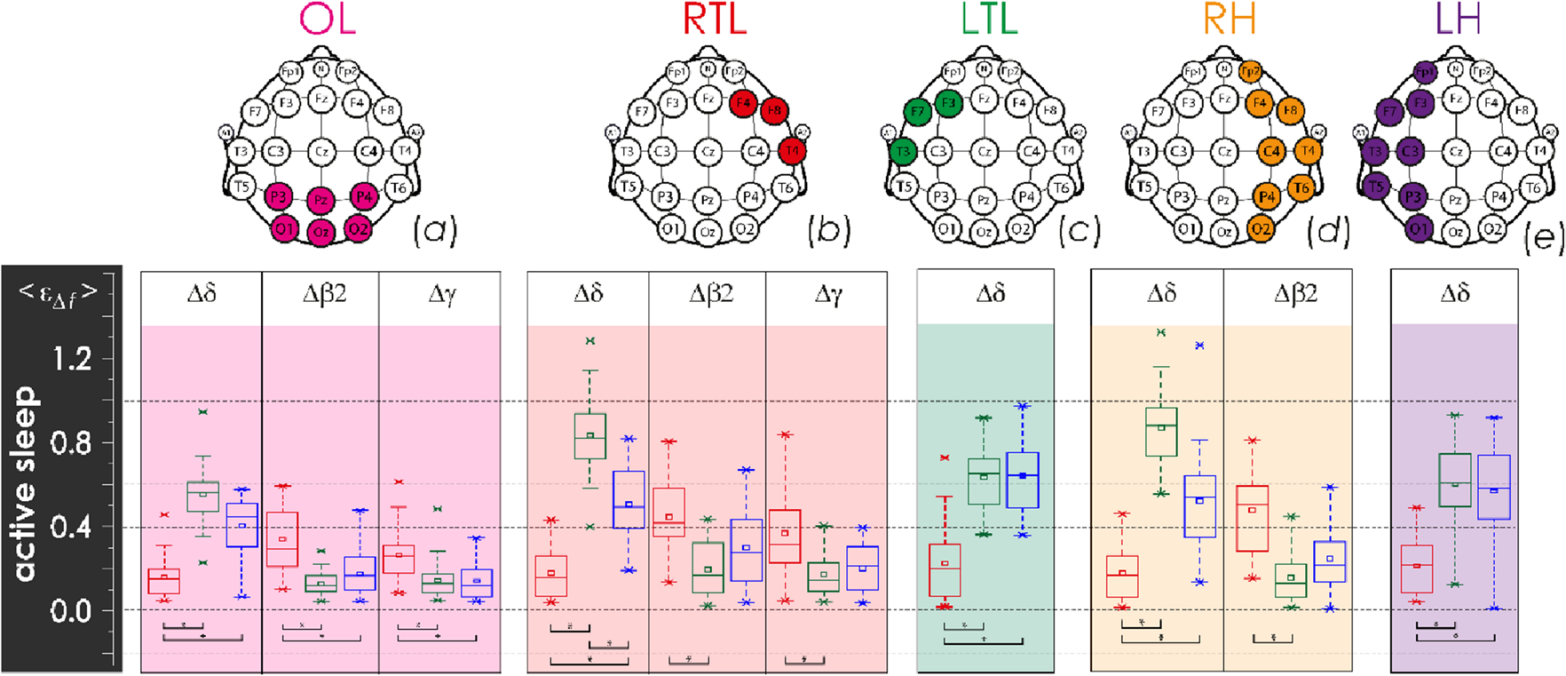
Distributions of the integral averages of relative CWT amplitudes, ${\langle {\upvarepsilon }_{\Delta \mathrm{f}}\rangle }_{\mathrm{QS}},$ based on EEG monitoring in newborn groups I and II for the various lobes of the brain, calculated under the physiological state of active sleep (AS): (**a**) diagrams for the occipital lobe (OL); (**b**) diagrams for the right temporal lobe (RTL); (**c**) diagrams for the left temporal lobe (LTL); (**d**) diagrams for the right hemisphere (RH); (**e**) diagrams for the left hemisphere (LH). The localization of EEG channels on the scalp for each brain lobe is shown above. Red color indicates estimates for group II (EEG monitoring event GA34–37^10d^), green and blue colors code the estimates of the first and second monitoring events in group I, GA38–41^8h^ and GA38–41^10d^, respectively. The diagrams depict the following statistical characteristics of numerical indicators: the first and the third quartiles (25–75%, inside the box), the median and the mean (transverse line and point inside the box, respectively), 1.5 interquartile ranges (shown by whiskers), and outliers represented by asterisks. At the bottom, underlining with asterisks highlights the pairs of values meeting the Mann–Whitney test with p < 0.001.

**Figure 3:**
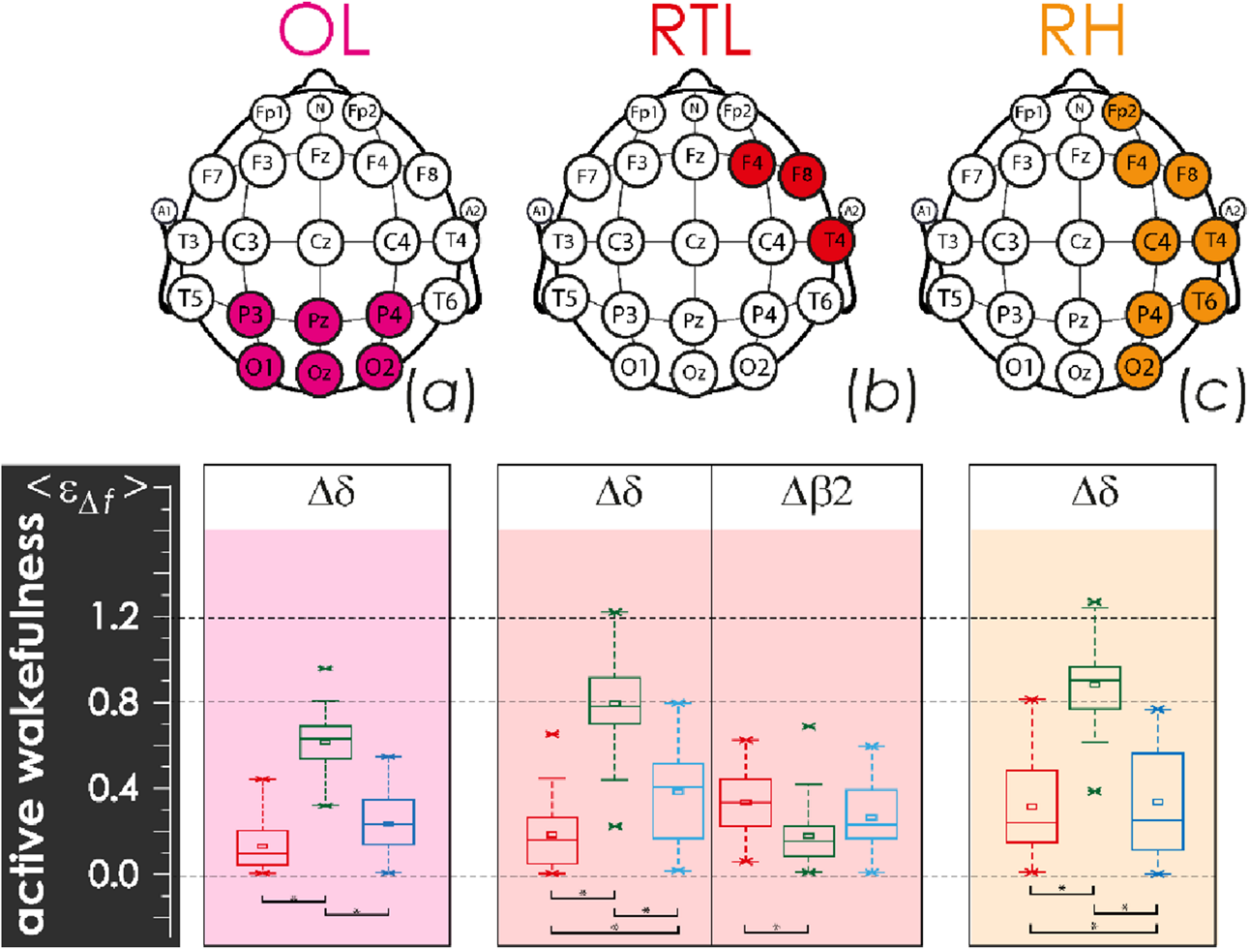
Distributions of the integral averages of relative CWT amplitudes, ${\langle {\upvarepsilon }_{\Delta \mathrm{f}}\rangle }_{\mathrm{QS}},$ based on EEG monitoring in newborn groups I and II for the various lobes of the brain, calculated under the physiological state of active wakefulness (AW): (**a**) diagrams for the occipital lobe (OL); (**b**) diagrams for the right temporal lobe (RTL); (**c**) diagrams for the left temporal lobe (LTL). The localization of EEG channels on the scalp for each brain lobe is shown above. Red color indicates estimates for group II (EEG monitoring event GA34–37^10d^), green and blue colors code the estimates of the first and second monitoring events in group I, GA38–41^8h^ and GA38–41^10d^, respectively. The diagrams depict the following statistical characteristics of numerical indicators: the first and the third quartiles (25–75%, inside the box), the median and the mean (transverse line and point inside the box, respectively), 1.5 interquartile ranges (shown by whiskers), and outliers represented by asterisks. At the bottom, underlining with asterisks highlights the pairs of values meeting the Mann–Whitney test with p < 0.001.

**Figure 4:**
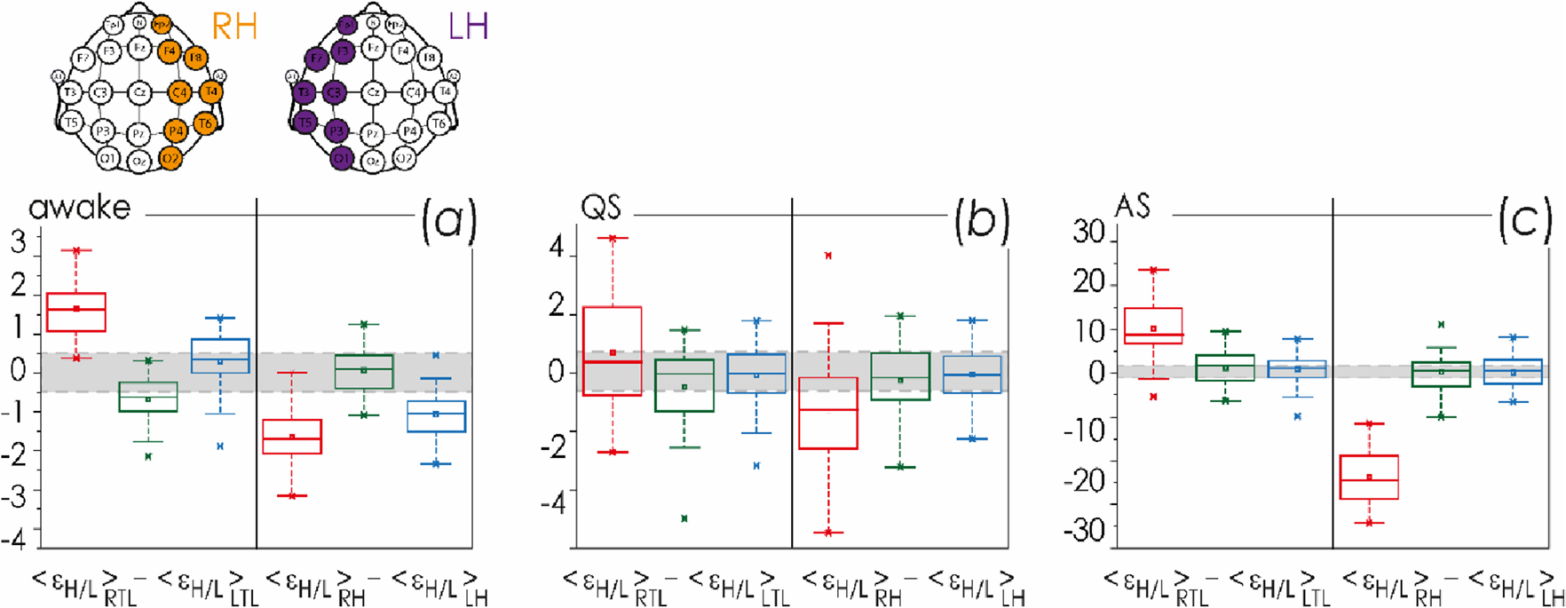
Distributions of the differences of integral energy ratios in the high frequency range to the low frequency range, ${\langle {\upvarepsilon }_{H}/L\rangle }_{\mathrm{AW},\mathrm{ AS},\mathrm{ QS}}$, between the right and left hemispheres, RH and LH: (**a**) diagrams for AW state; (**b**) diagrams for QS state; (**c**) diagrams for AS state. The localization of EEG channels on the scalp for each hemisphere is shown above. Red color indicates estimates for group II (EEG monitoring event GA34–37^10d^), green and blue colors code the estimates of the first and second monitoring events in group I, GA38–41^8h^ and GA38–41^10d^, respectively. Gray color shows the difference range [− 0.5; 0.5], corresponding to minor activity differences between the right and left hemispheres. The diagrams depict the following statistical characteristics of numerical indicators: the first and the third quartiles (25–75%, inside the box), the median and the mean (transverse line and point inside the box, respectively), 1.5 interquartile ranges (shown by whiskers), and outliers represented by asterisks.

**Figure 5:**
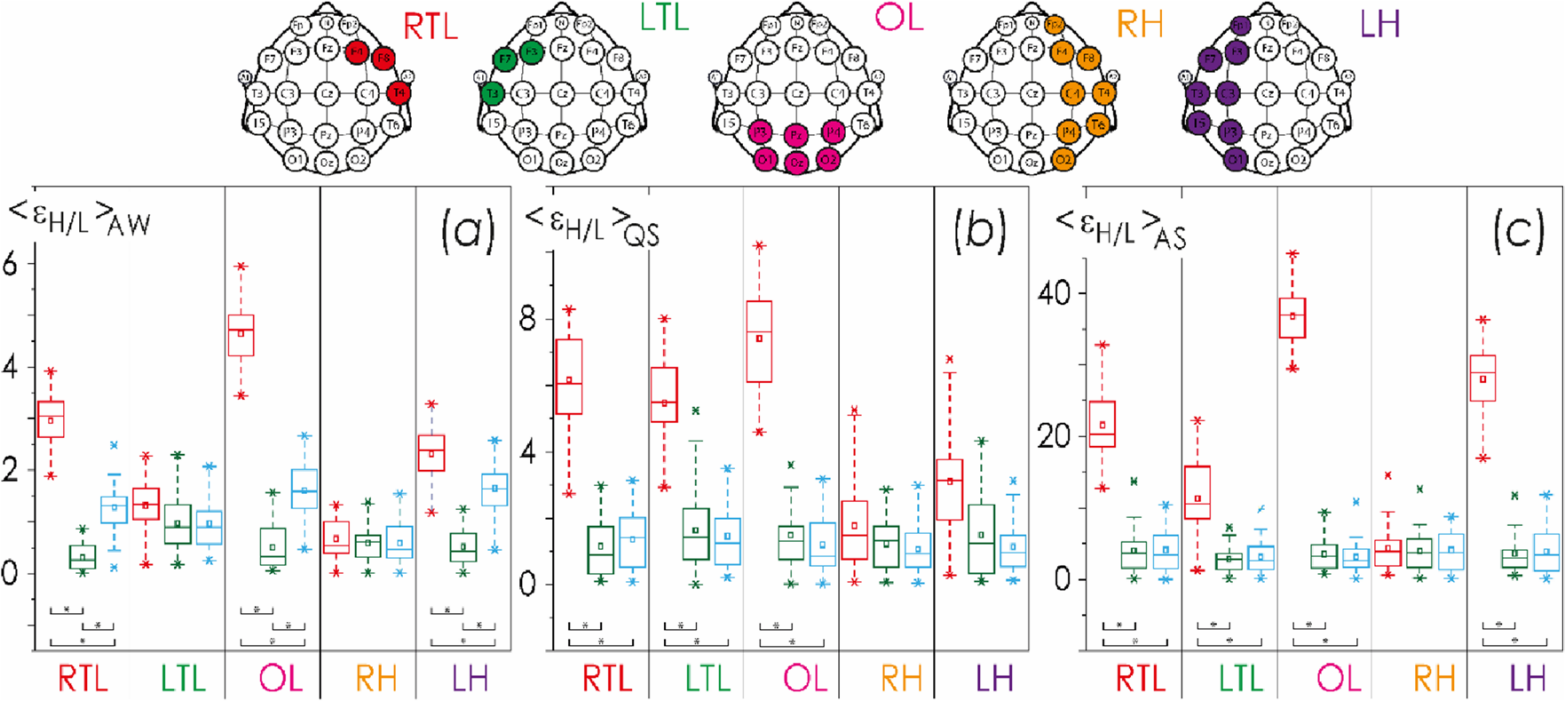
Distributions of the mean values of the integral energy ratios of the high frequency range to the low frequency range, ${\langle {\upvarepsilon }_{H}/L \rangle }_{\mathrm{AW},\mathrm{ AS},\mathrm{ QS}}$, for each brain region under consideration. These means are calculated for the physiological states of AW (**a**), QS (**b**) and AS (**c**). Acronyms for spatial zones are given under each diagram in accordance with Table 2. Red color indicates estimates for group I, GA34–37^10d^. Green and blue colors code the estimates for the first and second monitoring events (GA38–41^8h^ and GA38–41^10d^, respectively) in group II. The localization of EEG channels on the scalp for each hemisphere is shown above. The diagrams depict the following statistical characteristics of numerical indicators: the first and the third quartiles (25–75%, inside the box), the median and the mean (transverse line and point inside the box, respectively), 1.5 interquartile ranges (shown by whiskers), and outliers represented by asterisks. At the bottom, underlining with asterisks highlights the pairs of values meeting the Mann–Whitney test with p < 0.001.

In the spatial regions of the left and right temporal lobes and cerebral hemispheres, we estimated relative amplitudes of oscillatory activity based on fragments of EEG signals, according to different physiological states (QS, AS, AW). By averaging GA38–41^8h^, GA38–41^10d^ and GA34–37^10d^, we compared the observed characteristics of the oscillatory microstructure of sleep and wakefulness between groups I and II, and between GA38–41 and GA34–37, respectively. Figures 1, 2 and 3 reflect reliably detected differences in certain frequency ranges for various spatial regions of EEG brain activity recorded during the GA38–41^8h^ (green diagram), GA38–41^10d^ (blue diagram) and GA34–37^10d^ (red diagram) monitoring events. Full results of statistical assessments of integral averages of relative amplitudes calculated for various physiological states of newborns in groups I and II are presented in Figs. 1–5 in the Appendix.

The occipital lobe demonstrated the slowest activity (frequency range ∆δ) of EEG monitoring event GA34–37^10d^ in group II of late preterm infants who were characterized by lower values, as compared to the base group I (GA38–41^8h^, GA38–41^10d^). At the same time, full-term newborns demonstrated an increase in slow-wave activity during quiet sleep (Fig. 1a) and, simultaneously, a reduction in its level during active sleep (Fig. 2a) and wakefulness (Fig. 3a), observed when comparing the results of the first and second monitoring events, GA38–41^8h^ and GA38–41^10d^, respectively. On the contrary, the level of rapid oscillatory activity, characterized by the frequency ranges ∆β1 − ∆γ, increased during EEG monitoring event GA34–37^10d^ in group II, compared with group I (recordings GA38–41^8h^ and GA38–41^10d^) for physiological state of AS (see Fig. 2a). Similar processes of high-frequency activity growth in the occipital lobe in group II were also observed for other physiological conditions, as can be seen in Fig. 1 in the Appendix. However, these differences were not statistically significant. The oscillatory activity in high frequency ranges (∆β1, ∆β2, ∆γ) was state-independent in infants. Moreover, in newborns of group II (EEG monitoring event GA34–37^10d^), it took maximum values during the QS-state.

Overall, the right and left temporal lobes, RTL and LTL, demonstrated a minor increasing of slow oscillatory mode amplitudes, characterized by the ∆δ frequency range, prevailed in QS state of group I newborns, during the transition from GA38–41^8h^ monitoring event to GA38–41^10d^ (Fig. 1b). Rapid oscillatory activity of RTL in monitoring event GA34–37^10d^ in group II newborns was increased, compared with similar processes of the first and second monitoring events in group I. For states of active sleep and wakefulness, these differences were significant, as shown in Figs. 2b and 3b. The complete information is presented in in Fig. 2 of the Appendix. Besides, it is worth noting that over the first ten days of postnatal development, the RTL significantly reduced the activity of the slowest oscillatory mode (∆δ frequency range) during the states of active sleep and active wakefulness in group I, as reflected in Figs. 2b and 3b, respectively.

With greater averaging performed across the regions of the right and left hemispheres, RH and LH, the revealed patterns were less pronounced. In general, during the transition from the monitoring event GA38–41^8h^ to the monitoring event GA38–41^10d^ in group I, it was possible to observe insignificant changes of oscillatory activity in the ∆δ frequency range for sleep states, QS and AS, as shown in Figs. 1 and 2. However, EEG activity of the right hemisphere in the state of wakefulness has revealed significant reduction of low-frequency oscillations (Fig. 3). At the same time, the maturation of group I, observed during the transition from GA38–41^8h^ to GA38–41^10d^, was accompanied by minor changes in the rapid oscillatory activity of the frequency ranges ∆β2 and ∆γ for all physiological states.

**Figure 6:**
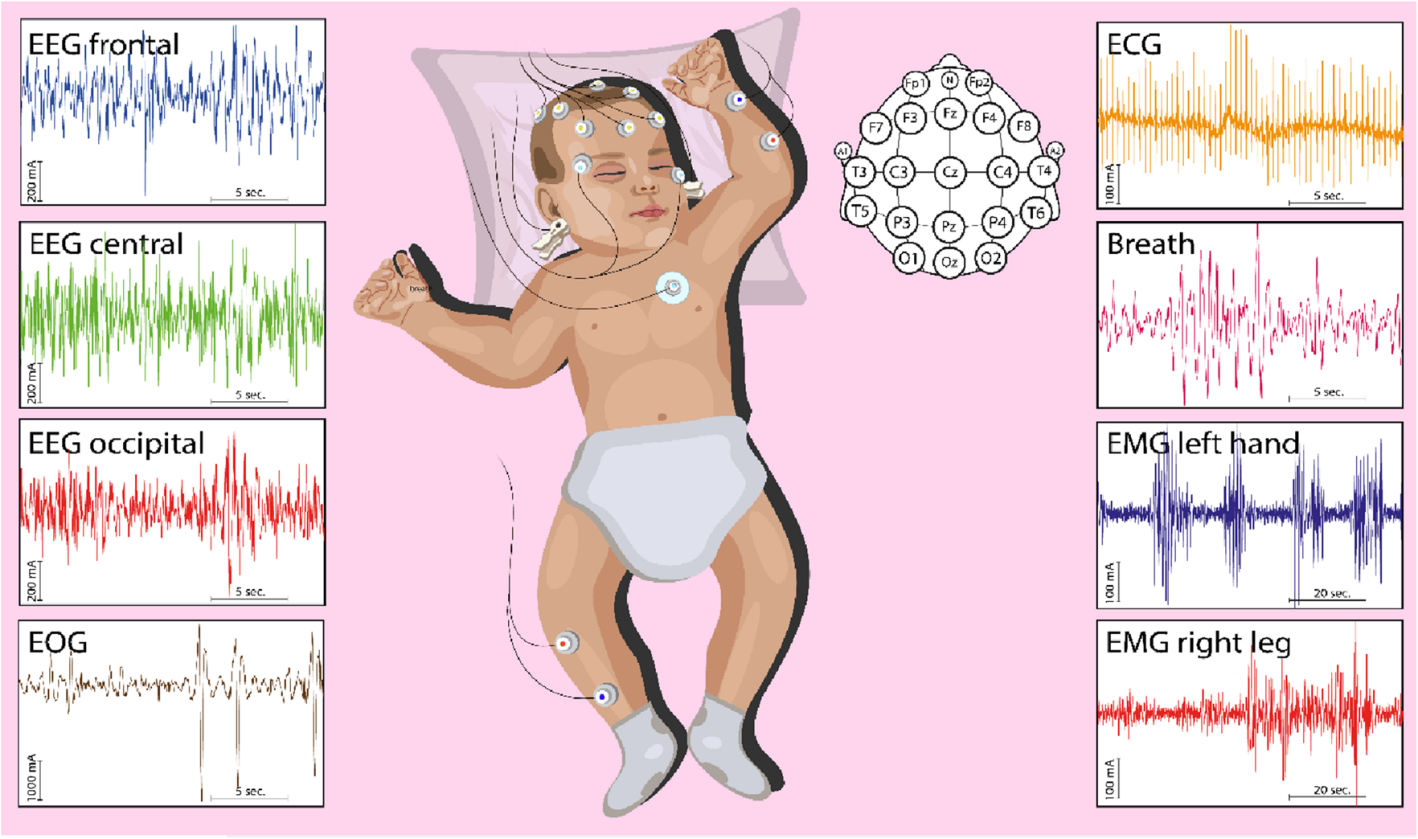
Newborn monitoring scheme, including EEG, EOG, ECG, EMG Left Arm, and EMG Right Leg. The diagram is supplemented with several fragments of EEG, ECG, EMG, and EOG recordings for one of the newborns in group I (with a gestational age of 40.3 weeks). For clarity, fragments of biomedical records in the figure are presented at different scales.

Analysis of oscillatory activity in σ range, [12; 15] Hz, did not reveal significant differences in the EEG activity of brain lobes for the analyzed recording events GA34–37^10d^, GA38–41^8h^ and GA38–41^10d^, as shown in Fig. 6 of the Appendix.

For all newborns, it was possible to observe a pronounced interhemispheric difference. In group I, the right hemisphere was characterized by uniform activity in the frequency ranges ∆θ, ∆α, ∆β1 and ∆γ, i.e., mean activity amplitude for these oscillatory modes was similar during active wakefulness and active sleep. The activity of the left hemisphere was significantly less homogeneous and more variable, which was especially well demonstrated by group II.

Besides, the oscillatory activity of the right hemisphere in group II was even more homogeneous: virtually identical amplitudes were observed during wakefulness and active sleep in all frequency ranges, except for ∆β2. A similar situation could also be observed during the QS state, with the difference that the variability was generally higher, and the maximum energy of the oscillatory modes fell on the ∆β1 and ∆β2 ranges. Also, the EEG activity of the left hemisphere of the brain during the state of wakefulness in group II was very similar to that in newborns of group I. However, the QS and AS states allowed easy distinguishing between groups I and II by oscillatory EEG activity in the ∆δ and ∆β2 frequency ranges.

In Fig. 4, we present the difference in the EEG activities of right and left hemispheres, RH and LH, based on the ratios of oscillatory activity in high and low frequency ranges, ${\langle {\upvarepsilon }_{H}/L\rangle }_{\mathrm{AW},\mathrm{AS},\mathrm{QS}}$.

Figure 5 presents the results of estimating the mean values of the integral amplitude ratios of the fast and slow oscillatory modes, ${\langle {\upvarepsilon }_{H}/L \rangle }_{\mathrm{AW},\mathrm{AS},\mathrm{QS}}$. The physiological state of active wakefulness makes it possible to distinguish between the newborns of all three subgroups, with the exception of averaging over the LTL and RH regions. At the same time, in the state of sleep, it is not possible to reliably distinguish the age difference of 10 days in full-term neonates. However, it is interesting that with a relatively ‘weak’ averaging (below the hierarchical level of hemispheres), for the QS state, it is possible to obtain a statistically significant difference between these neonates and those born a little earlier than 40 weeks of gestation; whereas for the AS state, such difference remains even when averaging over the entire left hemisphere.

## Discussion

Currently, the study of apparently normal nonconvulsive activity of the brain in newborns continues attracting considerable attention of researchers, mainly due to the need of developing reliable systems for detecting the state of sleep/wakefulness and searching for accurate criteria of early detection of neurological developmental disorders in newborns. However, most published studies of neonates were devoted to examining the first 3–5 weeks of their life^33^. From a clinical standpoint, the results of the analysis of changes at this age are of particular importance, since after the first week of life, especially in developing countries, not all parents are ready to come to the clinic for long-term monitoring and prognosis not associated with an immediate life threat. During the first days after birth, mother and newborn, being in the conditions of maternity ward, would react more indifferently to the safe and painless procedure of recording various physiological activity signals. In our study, we demonstrate the results of examining the EEG brain activity of newborns in the first days of their life (up to the age of 2 weeks).

Having considered relatively large groups of full-term and late preterm newborns, we concluded that even a small change in gestational age led to significant changes in the oscillatory characteristics of brain activity. Overall, these results expand the understanding of changes in the functional structure of brain activity connections, previously shown for extremely preterm neonates with a gestational age of 25–28 weeks in the study by Tokariev et al. ^34^.

An accurate time–frequency analysis of the brain activity in full-term newborns demonstrated the homogeneity of EEG time–frequency assessments during different stages of sleep in the course of two conducted monitoring sessions. In other words, all age-related frequency changes in the brain activity of newborns of apparently normal gestational age were inherent of the active wakefulness stage.

Our study results are characterized by already developed interhemispheric connections, observed in groups of newborns from the first days of their lives. We see that averaging the characteristics over the space of the right hemisphere leads to nearly uniform response of the brain in all considered physiological states of sleep and wakefulness. We can assume that for neural activity processes, occurring at different rates, the variability of functional connections creates the prerequisites for further development of the universal characteristics of human speech associated with activity networks of the left hemisphere in adults, regardless of their native language, as shown by Malik-Moraleda et al.^35^.

Of particular importance is the fact that children born a few weeks earlier than the usual gestational age demonstrate the following: (1) a significantly higher variability in the characteristics of the brain oscillatory activity, (2) statistically significant deviations from the EEG characteristics of full-term infants, and (3) significant interhemispheric differences. Based on the described age-related processes in full-term newborns, it can be assumed that the right hemisphere of the brain is overall slower and more rigid in terms of changes in the electrical dynamics of the cortex observed on the EEG. The small LTL region identified in the left hemisphere may, on the contrary, provide an example of the most rapid age-related changes in the first days of life, which leads to the absence of differences in wakefulness in this region, based on the analysis of all performed monitoring events. In other words, the development of functional patterns of brain activity for the wakefulness occurs at a faster rate than for the sleep states. The characteristic EEG patterns during sleep in mildly preterm infants seem to develop slower, resulting in significantly different time–frequency characteristics from those of full-term newborns.

We also emphasize definite presence of early developing alpha activity marker in the occipital region during the QS in infants born before the usual gestational age. Compared with full-term newborns, alpha activity demonstrates higher variability and amplitude. In addition, for nearly all spatial regions under consideration, alpha activity is more pronounced in premature newborns in the sleep state, which suggests the presence of a compensatory mechanism for its occurrence in the areas of primary and secondary sensory perception in the cerebral cortex. This finding requires further studying, along with a monitoring of the neurological development in a group of children during at least the first year of their lives.

Hence, a detailed analysis of the EEG activity in moderately preterm newborns suggests that the oscillatory activity of their brain has a somewhat greater amplitude and magnitude in various frequency ranges, as compared with the full-term newborns (both in the immediate postnatal period and after ten days of adaptation). These results are in good agreement with the studies of O’Toole, Kenosi, Koolen, and other researches^12,14,15,36,37^, which also implies that the period of postnatal development significantly affects the dynamics of brain characteristics. The results of the study suggest that the current development of automatic systems for distinguishing the sleep stages of newborns^23,33,34^, based on the analysis of the brain electrical activity, should also consider an extensive scatter of values in objective indicators of brain activity even in groups of newborns that are sufficiently homogenous in terms of their physical parameter values.

## Materials and methods

### Patients

Our experimental work was carried out in compliance with all required ethical standards^38^ and was approved by the Ethics Committee at Saratov State Medical University (SSMU), Saratov, Russia. All experiments were performed in accordance with relevant guidelines and regulations. Before the onset of the study, written informed consent for monitoring, subsequent mathematical processing of the data, and publication of the results was obtained from all parents (or legal guardians of participants). Parents were present during all experiments in compliance with the requirements of the Russian Federation legislation.

The experimental material was collected during a clinical study, which included 98 full-term and late premature newborns. The inclusion criteria for the study were: gestational age at birth over 33.5 weeks, birth weight over 1500 g, and voluntary informed consent signed by the parents of a newborn child. To improve the statistical significance of intergroup differences and to reduce an impact of lurking variables, solely naturally born children (i.e., without any surgical interventions) were included in our study. The exclusion criteria were as follows: grade 3 intraventricular hemorrhage with a breakthrough into the brain substance, presence of a genetic pathology, and gross congenital malformations. All newborns underwent a complete clinical examination in accordance with the neonatology standards for the provision of medical care (clinical examination by a neonatologist and neurologist, complete blood count, blood biochemistry, neurosonography and genetic testing).

For each newborn, information was provided that was obtained by a neonatologist during a direct examination and registration of the basic physical data (gestational age, weight at birth, and mother’s age). All newborns were divided among groups I (GA38–41) and II (GA34–37) based on their gestational age (38–41 and 34–36 weeks, respectively). Table 1 presents the mean, standard deviation, and ranges of the patient data for each group.

Each child in group I underwent a noninvasive and painless functional monitoring procedure twice: at 8 h and 10 days after the birth. For the convenience of interpretation, we used the following notations for the first and second monitoring recordings, GA38–41^8h^ and GA38–41^10d^, respectively. Newborns from group II, specified as GA34–37^10d^, underwent similar monitoring procedure only on day 10 after birth. During the monitoring, each child was located in the crib. Monitoring of newborns was carried out at the clinical departments for newborns within the framework of scientific and clinical cooperation with SSMU.

The data of multichannel noninvasive monitoring of biomedical signals (EEG, EOG, ECG, EMG Left Arm, EMG Right Leg) were recorded using the Encephalan-EEGR-19/26 electroencephalograph (Medicom MTD LLC, Russia). All signals were sampled at 512 Hz and digitized at 16 bits for offline analysis using a personal computer. EEGs were obtaned via conventional monopolar recording method with two reference points and *N* = 20 electrodes arranged according to the 10–20 pattern^38,39^. Signals were recorded using Ag/AgCl electrodes in pre-mounted head units. Two reference electrodes, A1 and A2, were located on the mastoid processes, while the ground electrode N was placed above the forehead. The EEG signals were filtered by a band pass filter with cutoff points of 0.5 Hz and 40 Hz, and a notch filter of 50 Hz. ECG signals were used to evaluate heart rate parameters, and were analyzed using free software^40^. Figure 6 demonstrates a monitoring scheme, along with several fragments of EEG, ECG, EMG and EOG records.

The duration of monitoring session was 160–180 min. The records were supplemented by the protocol of a clinical research physician, describing in detail the infant’s condition (visually determined) and the activity mode (on the basis of a ‘manual’ analysis of heart rate indicators, etc.), such as wakefulness, anxiety, vocalization, active sucking, movements, active sleep (AS) and quiet sleep (QS)^41^. According to Bourel-Ponchel et al.^28^, among other publications, QS is characterized by lack of rapid eye movement (REM) sleep, a steady respiratory rate, the presence of a tonic chin on electromyogram (EMG), and few body movements, while AS is characterized by the presence of REM sleep, irregular respiratory rate, lack of tonic chin on EMG, and body and facial movements.

### Methods

To examine the time–frequency characteristics of electroencephalography, the traditional continuous wavelet transform (CWT) was used, which allowed estimating the power dynamics of oscillatory activity in different frequency ranges with a good time resolution^42^. The CWT, *W*_*i*_ (*f*, *t*), was calculated for each EEG signal, *x*_*i*_, based on the mother Morlet wavelet with the parameter Ω_0_ = 2π:

1 $${W}_{i}\left(f,t\right)=\sqrt{f}\underset{t-4/f}{\overset{t+4/f}{\int }}{x}_{i}\left(t\right){\left(\sqrt{f}{\pi }^{1/4}{e}^{j{\omega }_{0}f\left(t-{t}_{0}\right)}{e}^{\frac{{f\left(t-{t}_{0}\right)}^{2}}{2}}\right)}^{*}dt.$$

When using this value of the parameter Ω_0_, the CWT time scales can be represented in the conventional frequencies of the Fourier spectrum *f*, Hz^43^. The following processing algorithm was used to analyze the oscillatory activity of electroencephalograms.

**Figure 7:**
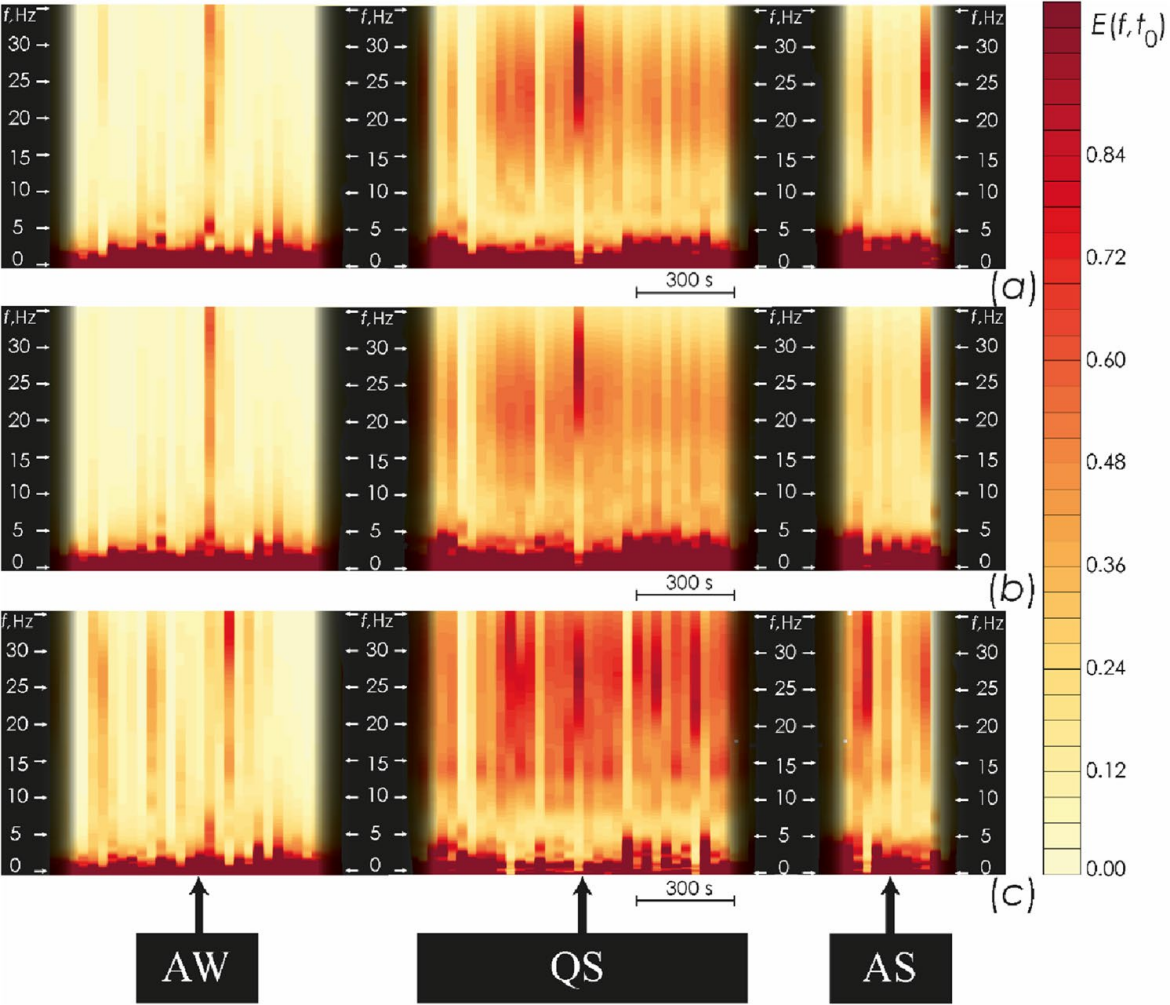
(**a–c**) Present the results of calculating the CWT energy, $E\left(f,{t}_{0}\right),$ averaged in the time window Δ*t* in different states—active wakefulness (AW), active sleep (AS) and quiet sleep (QS)—for the EEG channels O2, C4 and Fp2, respectively, recorded during the first monitoring session in a newborn # 7 from group I. The ordinate axes represent the frequencies of oscillatory activity (*f*, Hz), while the abscissa axes indicate time (*t*, s). The diagram shows a comparative time scale of 300 s. The color codes the mean amplitude of oscillatory activity, where red corresponds to the maximum value, while light yellow color depicts the minimum value.

1. For each EEG channel, the energy power *E* (*f*, *t*) was calculated for the frequency range [1.0; 40] Hz according to:
  2 $$E\left(f,t\right)= {\left|{W}_{i}\left(f,t\right)\right|}^{2}.$$
2. The energy power was averaged in the time window Δ*t* = 30 s as:
  3 $$E\left(f,{t}_{0}\right)= \frac{N}{\Delta t}\sum_{{t}_{1}}^{{t}_{2}}E\left(f,t\right),$$
  where (*t*_1_ = *t*_0_ − 0.5∙Δ*t*), (*t*_2_ = *t*_0_ + 0.5∙Δ*t*), and *N* represents the sampling step of the analyzed signal.
3. The energy power amplitudes for all channels were normalized to the maximum value for each time window, i.e., the energy value varied within [0; 1] conventional units. This method of integrated averaging makes it possible to emphasize the most significant oscillatory components of the brain electrical activity during each time window Δ*t*. Figure 7 shows an example of such dependences, *E* (*f*, ${t}_{0}$), for several EEG channels of one of the newborns. The map of *E* (*f*, ${t}_{0}$) values is located on the time–frequency plane: the abscissa shows the time (a discrete value that changes with a step Δ*t*) and the ordinate shows the frequency *f*, Hz.
4. For traditional frequency ranges, specifically delta, theta, alpha, beta1, beta2, gamma, and sigma, identified in accordance with currently accepted neurophysiological concepts^44^, the integral energy value, *E* (*f*, ${t}_{0}$), was estimated as follows:
  4 $${E}_{\Delta f}({t}_{0})={\sum }_{f\in \Delta f}E\left(f, {t}_{0}\right),$$
  where ∆ *f* was chosen from the values of the corresponding frequency ranges: ∆δ [1.0; 4], ∆θ [4; 8], ∆α [8; 12], ∆σ [12; 15], ∆β1 [15; 20], ∆β2 [20; 30], and ∆γ [30; 40] Hz.
5. Next, we performed normalization, assuming the total value of the CWT energies of all frequency ranges equal to one, $\sum_{\Delta f=\Delta \delta }^{\Delta \gamma }{E}_{\Delta f}({t}_{0})=1$. We also estimated the comparative shares of energy for each considered frequency range as:
  5 $$E\% _{{\Delta f}} (t_{0} ) = {{E_{{\Delta f}} (t_{0} )} \mathord{\left/ {\vphantom {{E_{{\Delta f}} (t_{0} )} {\sum\limits_{{\Delta f = \Delta \delta }}^{{\Delta \gamma }} {E_{{\Delta f}} } \left( {t_{0} } \right).}}} \right. \kern-\nulldelimiterspace} {\sum\limits_{{\Delta f = \Delta \delta }}^{{\Delta \gamma }} {E_{{\Delta f}} } \left( {t_{0} } \right).}}$$
6. As a result, we estimated mean energy of each oscillatory mode in the corresponding frequency range within several specific spatial regions of projections of certain cerebral cortex fields, specifically, the right and left temporal lobes (ε_∆*f*_|RTL and ε_∆*f*_|LTL) and occipital lobe (ε_∆*f*_ |OL), as well as the right and left hemispheres (ε_∆*f*_ |RH and ε_∆*f*_ |LH). Table 2 shows the EEG channels used to estimate mean energy for each region.

**Table 2:** Estimates of mean energy for oscillatory modes in the frequency ranges ∆δ, ∆θ, ∆α, ∆β1, ∆β2, and ∆γ for the scalp spatial regions of the right and left temporal lobes, and occipital lobe, as well as of the right and left hemispheres.

Region	Notation	EEG channels	Mean energy calculation algorithm
Left temporal	ε_∆*f*_|LTL	F7, F3, T3	${\upvarepsilon }_{\Delta f}({t}_{0})|\mathrm{LTL }=\frac{1}{3}\cdot \sum_{ch=\mathrm{F}7,\mathrm{F}3,\mathrm{T}3}{\left[{E\%}_{\Delta f}({t}_{0})\right]}_{ch}$
Right temporal	ε_∆*f*_|RTL	F8, F4, T4	${\upvarepsilon }_{\Delta f}({t}_{0})|R\mathrm{TL }=\frac{1}{3}\cdot \sum_{ch=\mathrm{F}8,\mathrm{F}4,\mathrm{T}4}{\left[{E\%}_{\Delta f}({t}_{0})\right]}_{ch}$
Occipital	ε_∆*f*_ |OL	P3, Pz, P4, O1, Oz, O2	$${\upvarepsilon }_{\Delta f}({t}_{0})|\mathrm{OL }=\frac{1}{6}\cdot \sum_{\begin{array}{c}ch=P3,Pz,P4,\\ O1,Oz,O2\end{array}}{\left[{E\%}_{\Delta f}({t}_{0})\right]}_{ch}$$
Left hemisphere	ε_∆*f*_ |LH	Fp1, F7, F3, T3, C3, T5, P3, O1	$${\upvarepsilon }_{\Delta f}({t}_{0})|\mathrm{LH }=\frac{1}{8}\cdot \sum_{\begin{array}{c}ch=Fp1,F7,F3,\\ T3,C3,T5,P3,O1\end{array}}{\left[{E\%}_{\Delta f}({t}_{0})\right]}_{ch}$$
Right hemisphere	ε_∆*f*_ |RH	Fp2, F8, F4, T4, C4, T6, P4, O2	$${\upvarepsilon }_{\Delta f}({t}_{0})|\mathrm{RH }=\frac{1}{8}\cdot \sum_{\begin{array}{c}ch=Fp2,F8,F4,\\ T4,C4,T6,P4,O2\end{array}}{\left[{E\%}_{\Delta f}({t}_{0})\right]}_{ch}$$

These spatial regions are shown in Fig. 1.

Additionally, the ratio of high and low frequency ranges of the oscillatory spectrum was calculated:

6 $$\varepsilon_{\tt{H\backslash L}}(t_0)=\frac{\sum_{\Delta f = \Delta\beta_1,\Delta\beta_2,\Delta\gamma}E\%_{\Delta f}(t_0)}{\sum_{\Delta f = \Delta\delta,\Delta\theta,\Delta\alpha}E\%_{\Delta f}(t_0)},\%.$$

**Figure 8:**
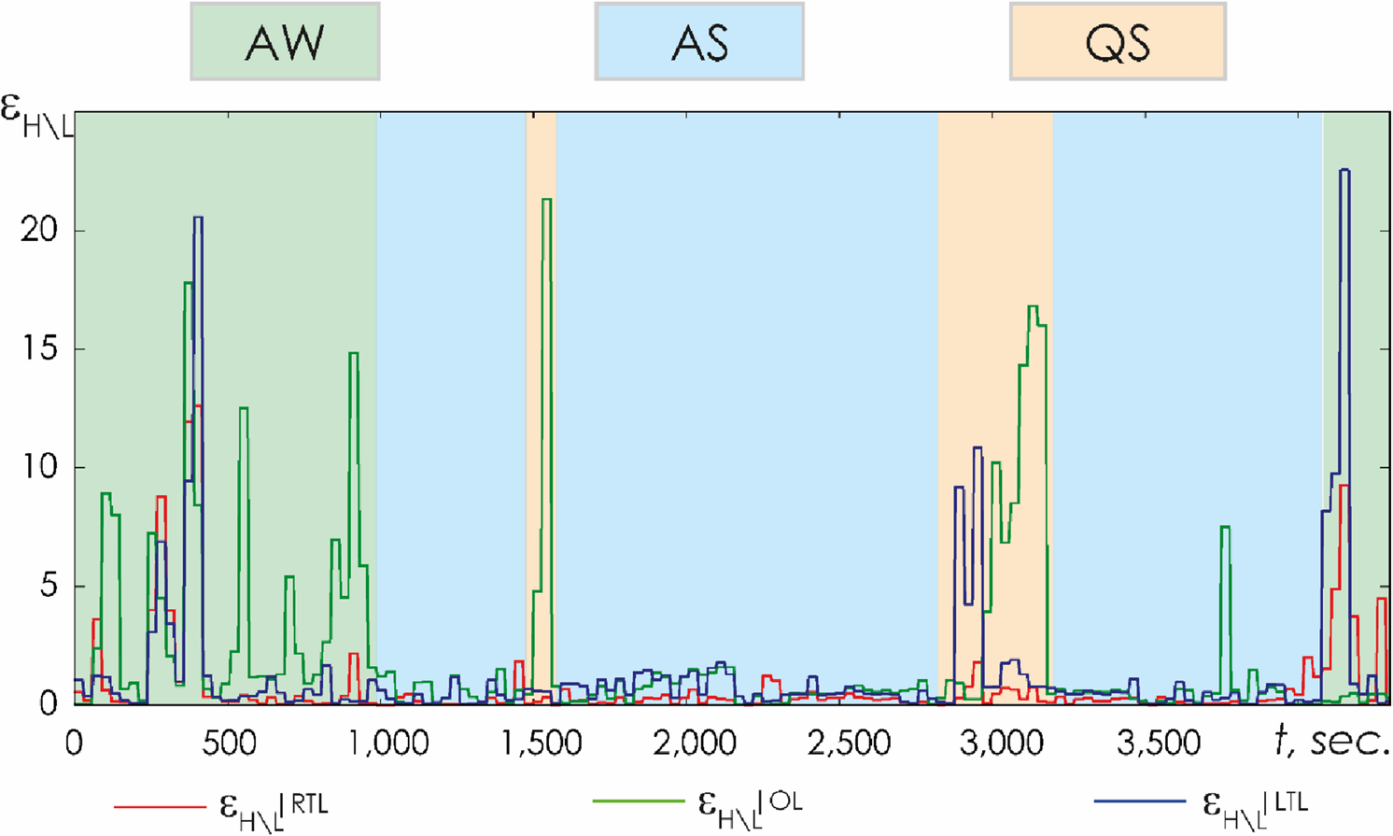
Relative energy calculation results for the right temporal (ε_H\L_|RTL, red line), occipital (ε_H\L_|OL, green line), and left temporal (ε_H\L_|LTL, blue line) lobes recorded during the second monitoring session in newborn # 42 from group I. The y-axis designates the value of the ratio (%); the x-axis represents time, *t* (s); the hypnogram for the newborn is presented as a color markup. Monitored stages are coded as follows: *AW* active wakefulness, *AS* active sleep, *QS* quiet sleep.

The ratio of the fast and slow oscillatory mode energies was similarly estimated both for each EEG channel and for considered spatial regions. Figure 8 illustrates this characteristic, calculated for the data recorded in one of the newborns of group I during the second monitoring event.

At the end of the processing, for each selected monitored stage (AW state, AS and QS states of sleep), the integral averages of relative amplitudes in each frequency range and each energy ratio of ‘fast’ and ‘slow’ oscillatory rhythms were estimated:

7 $${\langle {\upvarepsilon }_{\Delta \mathrm{f}}\rangle }_{\mathrm{AW},\mathrm{AS},\mathrm{QS}}=\sum_{{t}_{1}}^{{t}_{2}}{\upvarepsilon }_{\Delta f}(t)/{\Delta t}_{\mathrm{AW},\mathrm{AS},\mathrm{QS}},$$

8 $${\langle {\upvarepsilon }_{H}/L \rangle }_{\mathrm{AW},\mathrm{AS},\mathrm{QS}}=\sum_{{t}_{1}}^{{t}_{2}}{\upvarepsilon }_{\mathrm{H}\backslash \mathrm{L}}(t)/{\Delta t}_{\mathrm{AW},\mathrm{AS},\mathrm{QS}},$$

where AW, AS and QS subscripts denote the corresponding physiological states; *t*_1_ and *t*_2_ are the starting and ending times of registering this state, while ${\Delta t}_{\mathrm{AW},\mathrm{AS},\mathrm{QS}}$ is the duration of the state in question.

Mean, median, and standard deviation were used in descriptive statistics of the data. The Mann–Whitney U test for independent samples was used for the comparison of quantitative data^45^. The results with a p-value ≤ 0.001 were considered statistically significant. Statistical analyses were performed using the SPSS version 22.0 software for Windows (IBM, Armonk, NY, USA).

## Supplementary Information

Supplementary Figures.

## Data Availability

The datasets generated and/or analyzed during the current study are available from the corresponding author on reasonable request.
