# Supplementary material for: Examining time–frequency mechanisms of full-fledged deep sleep development in newborns of different gestational age in the first days of their postnatal development
Source: Sci Rep. 2022 Dec 14;12:21593. doi: 10.1038/s41598-022-26111-3 (PMC9751282; doi:10.1038/s41598-022-26111-3)
Supplement: Supplementary file 1 — Supplementary Figures. [file 41598_2022_26111_MOESM1_ESM.docx]

**
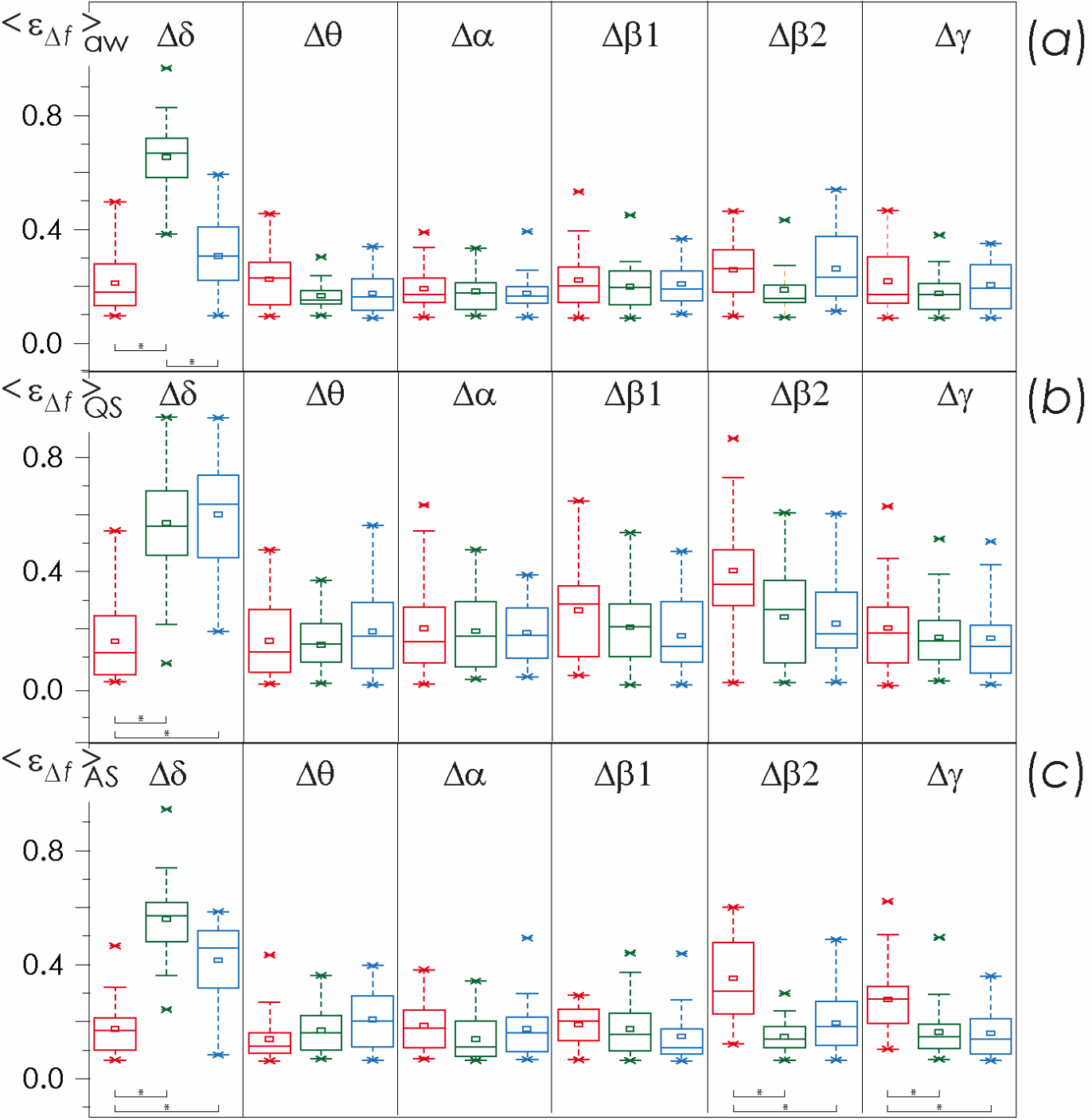
**

**Figure 1**. Distributions of the integral averages of relative CWT amplitudes, $\left\langle\varepsilon_{\Delta f} \right\rangle_{AW,AS,QS},$ in each group of newborns for the occipital lobe (OL) of the brain, calculated in the physiological states of active wakefulness – AW (*a*), quiet sleep – QS (*b*) and active sleep – AS (*c*). Red color designates estimates for group II, green and blue colors code the estimates of the first and second monitoring events in group I, respectively. The diagrams depict the following statistical characteristics of numerical indicators: the first and the third quartiles (25 – 75%, inside the box), the median and the mean (transverse line and point inside the box, respectively), 1.5 interquartile ranges (shown by whiskers), and outliers represented by asterisks.

**
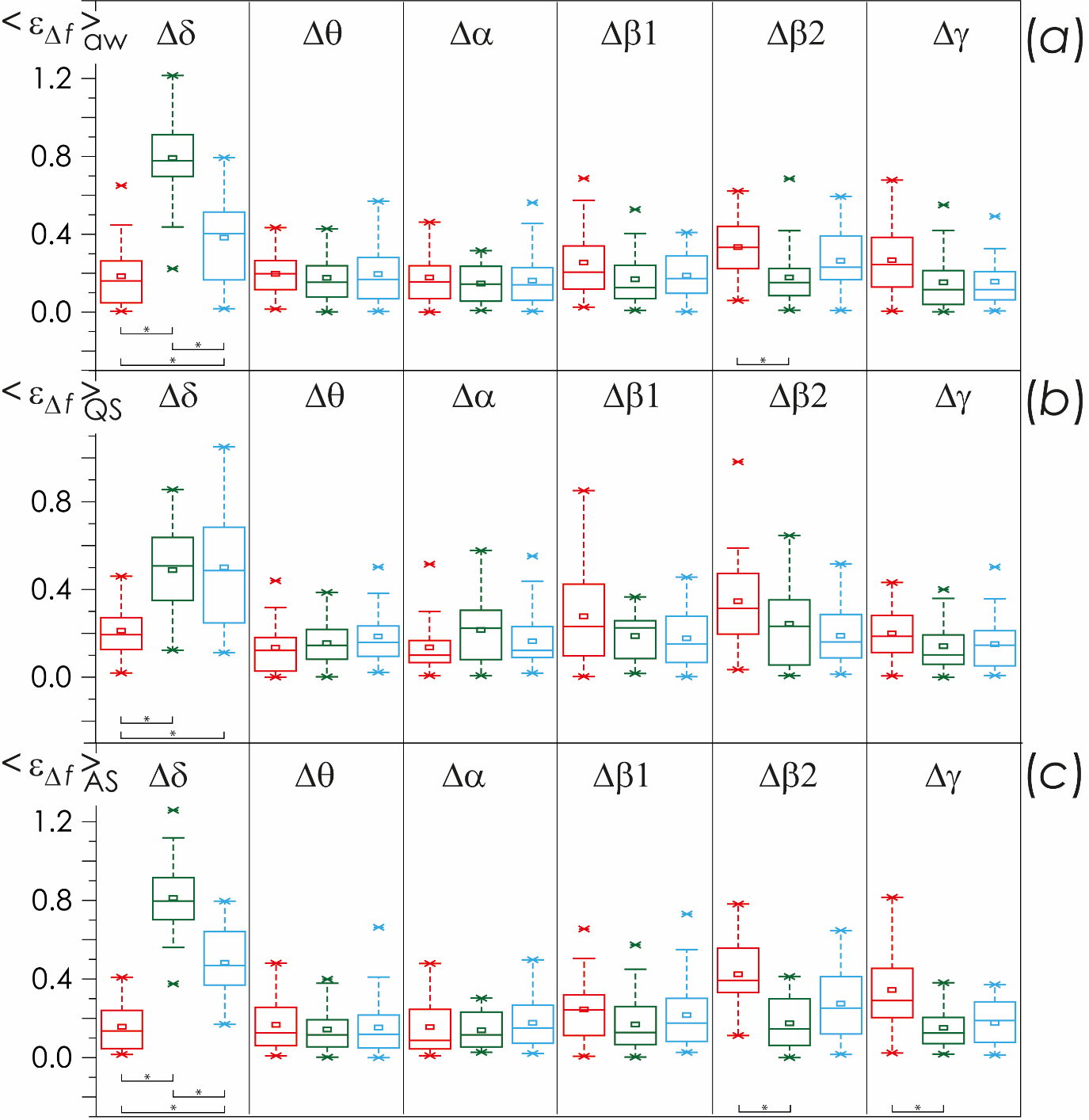
**

**Figure 2**. Distributions of the integral averages of relative CWT amplitudes, $\left\langle\varepsilon_{\Delta f} \right\rangle_{AW,AS,QS},$ in each group of newborns for the right temporal lobe (RTL) of the brain, calculated in the physiological states of active wakefulness – AW (*a*), quiet sleep – QS (*b*) and active sleep – AS (*c*). Red color designates estimates for group II, green and blue colors code the estimates of the first and second monitoring events in group I, respectively. The diagrams depict the following statistical characteristics of numerical indicators: the first and the third quartiles (25 – 75%, inside the box), the median and the mean (transverse line and point inside the box, respectively), 1.5 interquartile ranges (shown by whiskers), and outliers represented by asterisks.

**
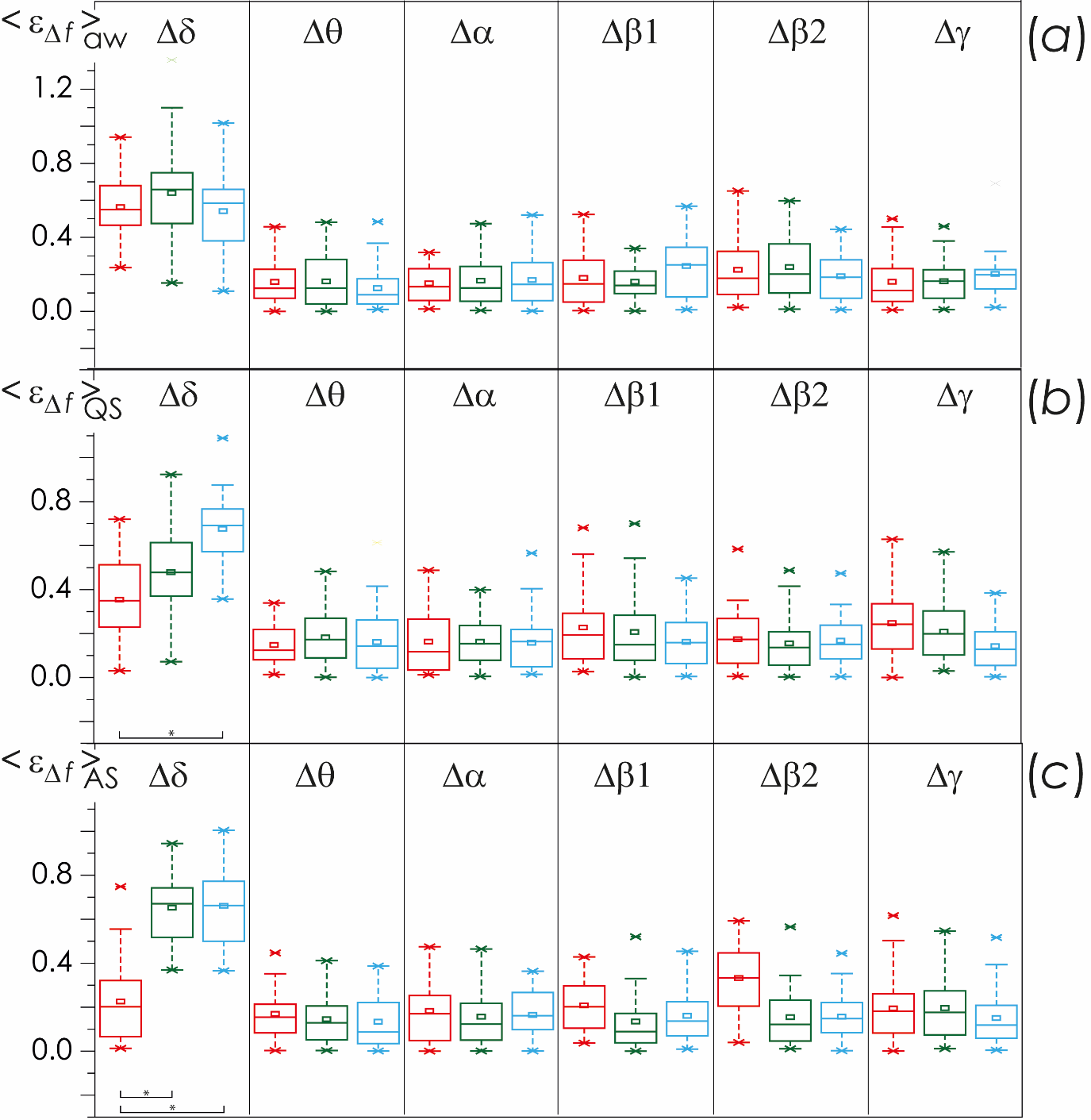
**

**Figure 3.** Distributions of the integral averages of relative CWT amplitudes, $\left\langle\varepsilon_{\Delta f} \right\rangle_{AW,AS,QS},$ in each group of newborns for the left temporal lobe (LTL) of the brain, calculated in the physiological states of active wakefulness – AW (*a*), quiet sleep – QS (*b*) and active sleep – AS (*c*). Red color designates estimates for group II, green and blue colors code the estimates of the first and second monitoring events in group I, respectively. The diagrams depict the following statistical characteristics of numerical indicators: the first and the third quartiles (25 – 75%, inside the box), the median and the mean (transverse line and point inside the box, respectively), 1.5 interquartile ranges (shown by whiskers), and outliers represented by asterisks.

**
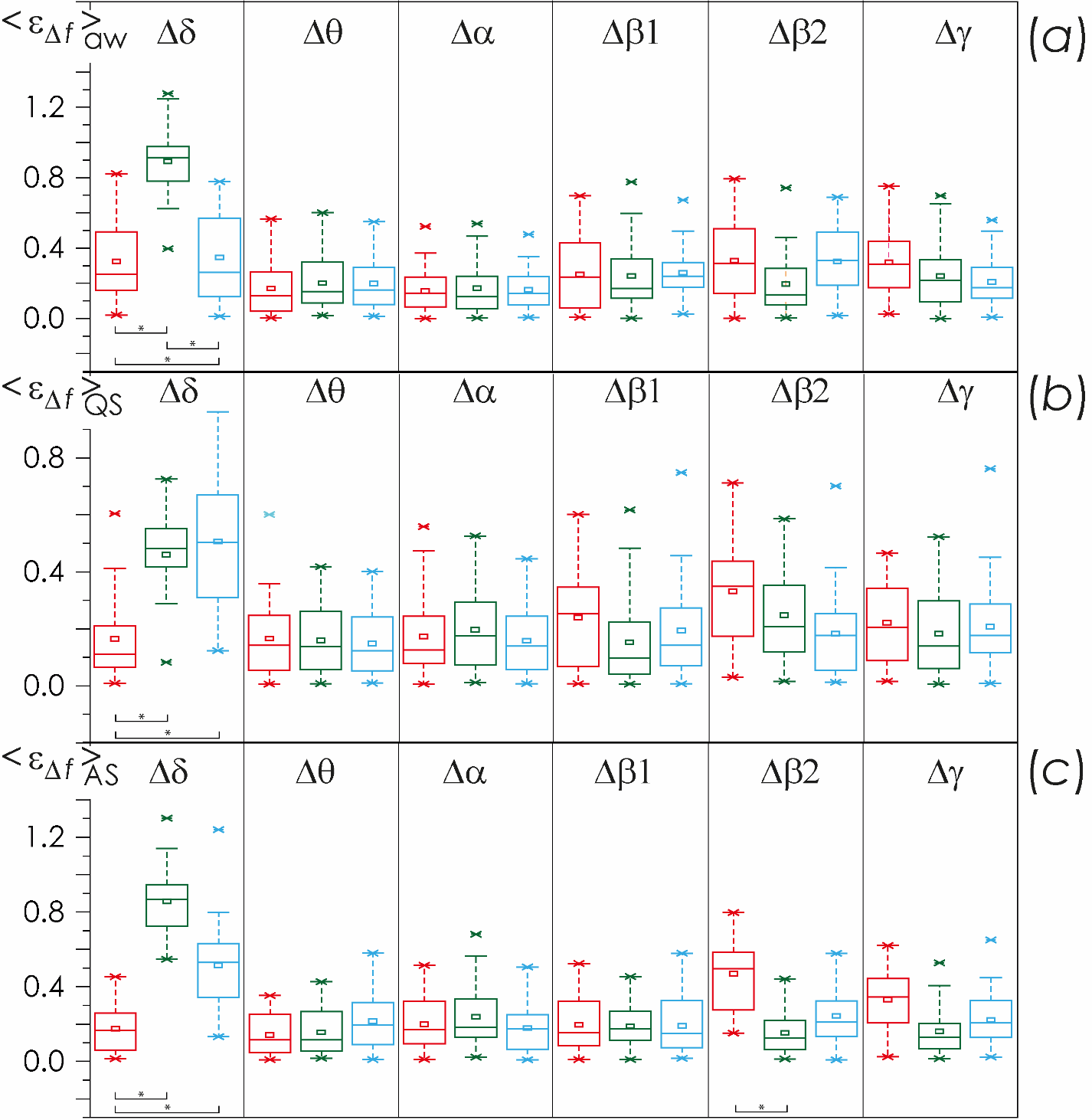
**

**Figure 4**. Distributions of the integral averages of relative CWT amplitudes, $\left\langle\varepsilon_{\Delta f} \right\rangle_{AW,AS,QS}$, in each group of newborns for the right hemisphere (RH) of the brain, calculated in the physiological states of active wakefulness – AW (*a*), quiet sleep – QS (*b*) and active sleep – AS (*c*). Red color designates estimates for group II, green and blue colors code the estimates of the first and second monitoring events in group I, respectively. The diagrams depict the following statistical characteristics of numerical indicators: the first and the third quartiles (25 – 75%, inside the box), the median and the mean (transverse line and point inside the box, respectively), 1.5 interquartile ranges (shown by whiskers), and outliers represented by asterisks.

**
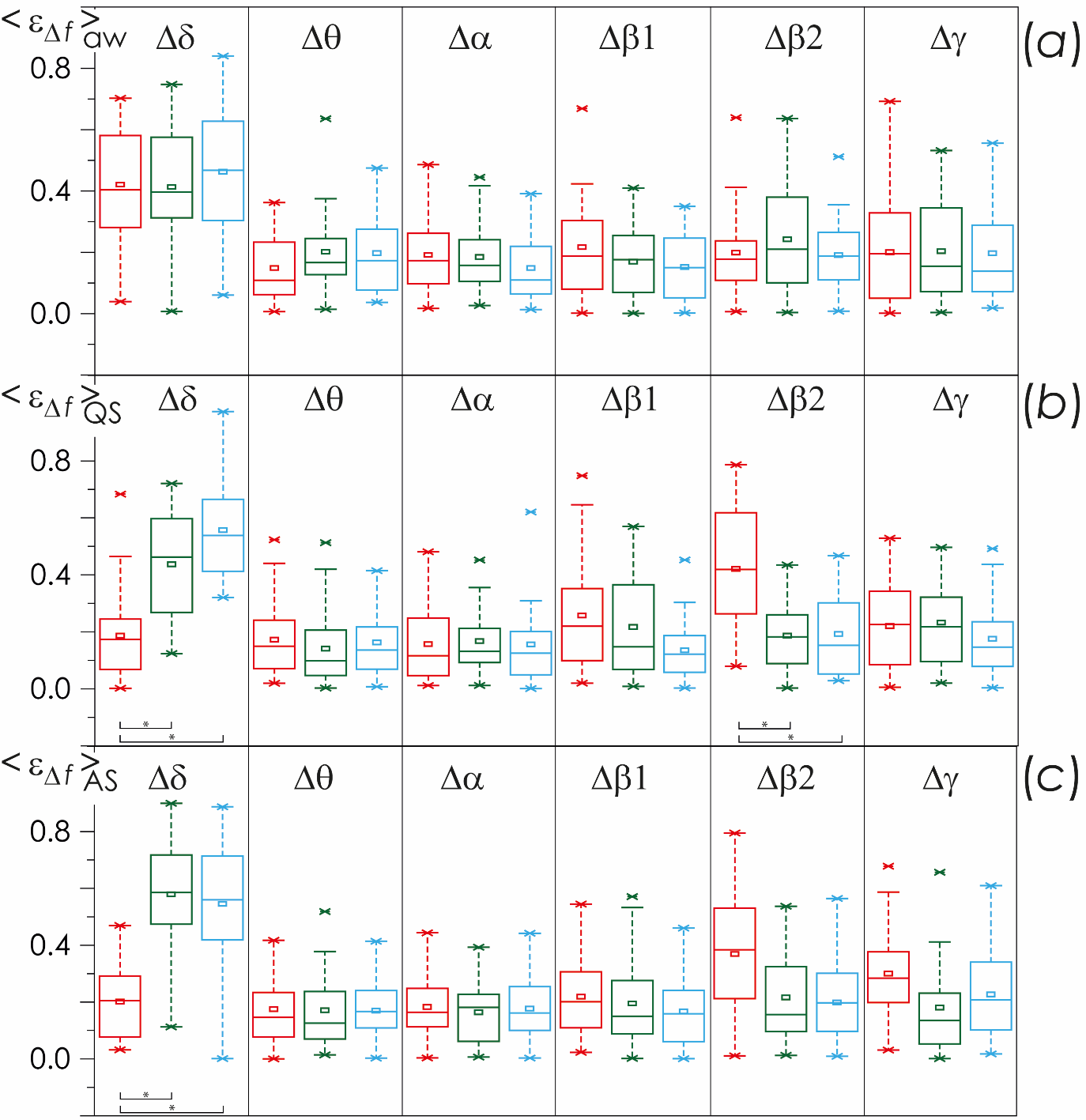
**

**Figure 5**. Distributions of the integral averages of relative CWT amplitudes, $\left\langle\varepsilon_{\Delta f} \right\rangle_{AW,AS,QS}$, in each group of newborns for the left hemisphere (LH) of the brain, calculated in the physiological states of active wakefulness – AW (*a*), quiet sleep – QS (*b*) and active sleep – AS (*c*). Red color designates estimates for group II, green and blue colors code the estimates of the first and second monitoring events in group I, respectively. The diagrams depict the following statistical characteristics of numerical indicators: the first and the third quartiles (25 – 75%, inside the box), the median and the mean (transverse line and point inside the box, respectively), 1.5 interquartile ranges (shown by whiskers), and outliers represented by asterisks.


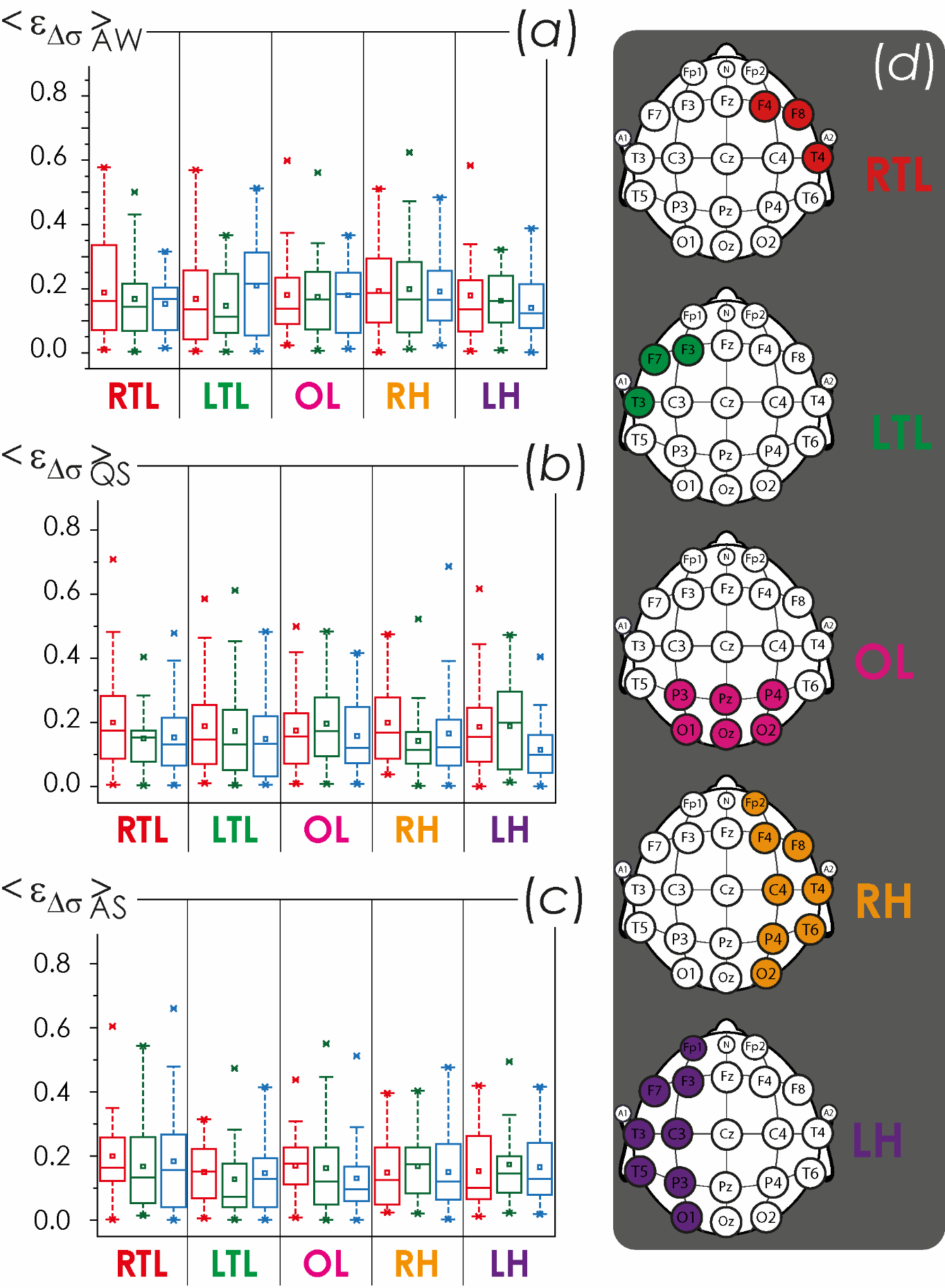


**Figure 6**. Distributions of the integral averages of relative CWT amplitudes, $\left\langle\varepsilon_{\Delta\sigma} \right\rangle_{AW,AS,QS}$, in each group of newborns for the left hemisphere (LH) of the brain, calculated in the physiological states of active wakefulness – AW (*a*), quiet sleep – QS (*b*) and active sleep – AS (*c*). Red color designates estimates for group II, green and blue colors code the estimates of the first and second monitoring events in group I, respectively. The diagrams depict the following statistical characteristics of numerical indicators: the first and the third quartiles (25 – 75%, inside the box), the median and the mean (transverse line and point inside the box, respectively), 1.5 interquartile ranges (shown by whiskers), and outliers represented by asterisks. On the right (*d*), the diagrams depicting the scalp location of indicated spatial regions of EEG activity are shown.
